# Estimating the likelihood of hospitalists to repeatedly prescribe high rates of antibiotics

**DOI:** 10.1017/ash.2025.10132

**Published:** 2025-09-18

**Authors:** Radhika Prakash Asrani, Samuel Parks, Chad Robichaux, K. Ashley Jones, Kristen Paciullo, Jesse T. Jacob, Shabir Hasan, Sujit Suchindran, Lucy S. Witt, Scott Fridkin

**Affiliations:** 1 Department of Medicine, Division of Infectious Diseases, Emory University School of Medicine, Atlanta, GA, USA; 2 Department of Biomedical Informatics, Emory University School of Medicine, Atlanta, GA, USA; 3 Emory Healthcare, Atlanta, GA, USA; 4 Emory Healthcare, Atlanta, GA, USA; 5 Division of Hospital Medicine, Department of Medicine, Emory University School of Medicine, Atlanta, GA, USA

## Abstract

Among 70 hospitalists across three facilities, 47% of high prescribers of broad-spectrum hospital-onset (BSHO) agents remained high in the subsequent period versus 24% for initially high prescribers of anti-MRSA agents. Findings of persistence of high prescribing add credibility to our metric for BSHO agents but not anti-MRSA agents.

## Introduction

Drug utilization reviews to improve antibiotic prescribing in the inpatient setting is labor intensive; metric driven tools are mostly limited to reporting of location-specific usage rates to the National Healthcare Safety Network (NHSN).^1,2^ While these metrics are valuable for monitoring usage, they lack adequate risk adjustment and ability to attribute prescribing to specific providers.^3^ While provider-specific metrics for peer-comparison to motivate behavior change have been proven effective in the ambulatory setting, technical challenges make similar activities in the inpatient setting scarce.^4,5^

While patients, providers, and payors have been increasingly asking for physician benchmarks for quality improvement, effective metrics must be credible to providers to influence their behavior.^6,7^ The reliability of quality measures considers variance as well as persistence.^8,9^ To improve credibility of an Emory Healthcare pilot program benchmarking inpatient antibiotic prescribing among hospitalists, we assessed whether providers with high prescribing metrics repeatedly prescribe high without intervention.^10^

## Methods

### Design and study population

We conducted a retrospective cohort study of antibiotic prescribing patterns using administrative data from three Atlanta hospitals from January–December 2023. Hospital A is a 582-bed comprehensive academic medical center; B and C are 537-bed and 373-bed specialized complex medical centers. All utilize dedicated hospital medicine services providing general medical care across multiple locations. Antibiotics administered to patients on dates billed by specific hospital medicine attendings were attributed to the billing attending, regardless of emergency medicine initiation or consultation involvement.

For each hospitalist, we used billing data to identify patient encounter dates and calculated billed patient-days (bPD). We extracted antibiotic prescribing data from electronic medication administration records (eMAR). For each bPD, matching transaction dates with NHSN-defined BSHO group antibacterial orders were captured as days of antibiotic therapy (DOT). Patient characteristics included demographics, age, microbiology results, ICD-10 antibiotic indications (pneumonia, COVID-19, sepsis, UTI), and comorbidities for Charlson score calculation. The methodology to derive the prescribing metric, an observed-to-expected ratio (OER), has been described elsewhere.^10^ Data were aggregated into bi-monthly intervals for each provider to reduce data sparsity from variable work schedules and minimizing imprecision for providers with less frequent billing practices. OERs were generated for NHSN-defined antibiotic groups: broad-spectrum hospital-onset (BSHO, mostly anti-pseudomonal agents) and anti-methicillin-resistant Staphylococcus aureus (Anti-MRSA).

### Analytic approach

**Figure 1.**
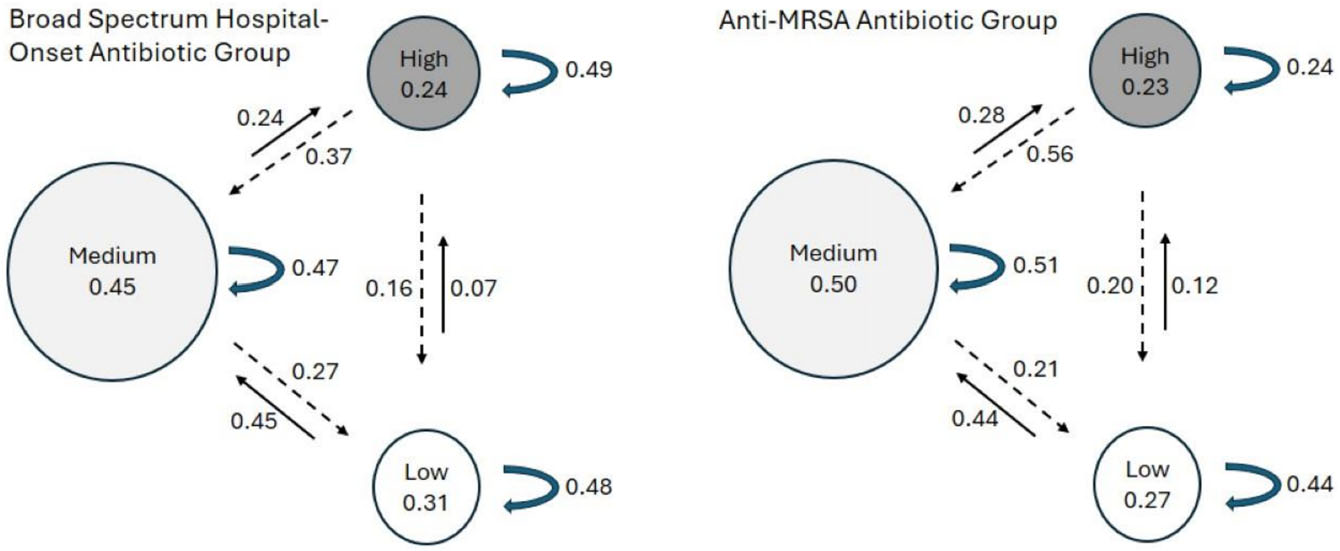
Markov Chain Plot, long-term probabilities (text within circles) of metric being high (OER > 1.25), medium (0.75 ≤ OER ≤ 1.25), or low (OER < 0.75), and immediate transition probabilities of metric change between subsequent periods progressing to higher state (solid arrow), lower state (dashed arrow), or remaining in same state (curved arrow), by antibiotic category.

Variance of BSHO and Anti-MRSA OERs were evaluated among providers at each hospital; roughly 25% of the providers had OERs >1.25 (25% prescribing > predicted). We employed two analytic approaches to evaluate persistence in prescribing behavior. First, we constructed transition matrices (Markov chain modeling) estimating immediate transition probabilities between any pair of sequential periods across three prescribing categories: low (OER <0.75), medium (OER 0.75–1.25), and high (OER >1.25), representing lowest quartile, interquartile range, and highest quartile, respectively. The immediate transition probabilities between states are represented by the values along each arrow in Figure 1. Steady-state probabilities were also calculated reflecting long-term probability (as system approaches infinite) the providers would remain in each prescribing state (values in each circle in Figure 1).

Second, we used log-binomial generalized estimating equations (GEE) quantifying associations between high prescribing in prior periods and subsequent periods. Models accounted for repeated measures and clustering at provider and facility levels. Separate models were estimated for BSHO and anti-MRSA agents, adjusting for sex and years since graduation as proxy for age and experience. Analyses used R Statistical Software (v4.2.0).

## Results

Across the three hospitals, 70 hospitalists (32, 45% at A; 20, 29% at B; 18, 26% at C) contributed data to six bi-monthly OERs during the 12–month study period resulting in 420 bi-monthly observations; this created 350 transitions of reporting metrics from one period to the next period. Per period, providers cared for a mean 132 patients (range 63–306) over 329 patient-days (range 165–478). Providers prescribed BSHO antibiotics at a mean 110 DOT per 1 000 patient-days (range 50–200) resulting in a mean OER of 0.96 (range 0.60–1.56), with provider-level variance in O:E ratios ranging from 0.29 to 1.63 (mean: 0.83). For Anti-MRSA antibiotics, providers prescribed a mean 86 DOT per 1 000 patient-days (range 9–191) resulting in a mean OER of 1.0 (range 0.12–2.34), with provider-level variance in O:E ratios ranging from 0.10 to 1.49 (mean: 0.80).

Markov chain modeling revealed long-term probabilities for providers to prescribe in each OER category. Hospitalists spend approximately half the time in the medium state with remaining time split between high and low prescribing states (Figure 1, circles). Long-term probabilities were similar between antibiotic groups: providers most often had OERs in middle category (45% and 50% for BSHO and anti-MRSA, respectively), then lowest category (31% and 27%), then highest category (24% and 23%). For immediate transitions, most providers remained in the same category (Figure 1, curved arrow). These probabilities were similar between BSHO and anti-MRSA agents at roughly 50% (range 44%–51%). However, for BSHO agents the likelihood of remaining at a high OER between subsequent periods was 49% more than double the 24% for anti-MRSA agents (Figure 1, curved arrows). The probabilities to move progressively up from a lower OER category were similar between antibiotic groups: 7–12% from low OERs to high OERs and 44–45% from low OERs to medium OERs (Figure 1, solid arrows). The probabilities to move progressively down from a higher OER to a lower OER was also similar between antibiotic groups (Figure 1, dashed arrows).

Log-binomial GEE regression models quantified persistence of high prescribing behavior. High BSHO prescribers were three times more likely to prescribe high in the next period compared to other prescribers (adjusted relative risk 3.72, 95% CI: 2.45–5.65), controlling for years since graduation and sex (Table). This indicates strong temporal persistence for BSHO agents. In contrast, for anti-MRSA agents, this persistence was not observed (adjusted rate ratio 0.46, 95% CI: 0.23–0.94).

## Discussion

Hospital medicine providers prescribing high amounts of BSHO antibiotic agents during one period are likely to continue prescribing high rates subsequently; however, this did not hold for anti-MRSA prescribing. This adds credibility to our BSHO OER as an internal performance metric. Specifically, persistence of “high” prescribing suggests chance alone does not adequately explain providers receiving high rates in any period. While the overall likelihood for any provider to prescribe high was only 24%, when in that category, there was roughly 50% chance of remaining there in the next period versus only 25% for anti-MRSA agents. We interpret these findings to mean general provider behavior influences BSHO prescribing and does not greatly vary over 4–month periods. Conversely, other factors outside provider behavior influence anti-MRSA prescribing as providers move in and out of high prescribing categories more often. Potential influencing factors include MRSA nasal screening swabs, which providers are encouraged to use for ruling out MRSA pneumonia; these results dramatically affect anti-MRSA de-escalation decisions. These data suggest providers prescribing high amounts of BSHO antibiotics will likely continue this pattern without intervention, creating opportunities for infectious disease pharmacist engagement or individual reflection on de-escalation. However, receiving high prescribing values doesn’t equate with inappropriate prescribing. We lack sustainable processes for appropriate diagnosis or treatment review. Additionally, patient factors affecting BSHO may not be fully captured by our OER calculations.^10^

Despite limitations, this metric is informative for targeting providers with high BSHO usage, suggesting their practices don’t occur simply by chance. As inpatient quality efforts and performance feedback metrics gain strength, evaluating attributes of potential performance metrics, such as persistence, should be added to considerations of reliability, accuracy, and clinical credibility.^7,9^

**Table 1.** Association between current high prescribing (OE ratio >1.25) and subsequent high prescribing using log-binomial GEE regression models for broad-spectrum hospital-onset and anti-MRSA antibiotics

Antibiotic group model	Risk ratio*	95% CI
Broad-Spectrum Hospital Onset Antibiotics	3.69	2.43, 5.62
Anti-MRSA Antibiotics	0.46	0.23, 0.94

* Adjusted for sex and years since a provider graduated from medical school, accounting for clustering by facility and provider.

## Research transparency and reproducibility

The datasets generated and/or analyzed for the current study are to be available in the Harvard Dataverse (link: https://dataverse.harvard.edu/).

## References

[1] Fridkin SK , Srinivasan A. Implementing a strategy for monitoring inpatient antimicrobial use among hospitals in the United States.Clin Infect Dis 2014;58:401–406. 24162744 10.1093/cid/cit710 PMC4645276

[2] Davey P , Marwick CA , Scott CL , et al. Interventions to improve antibiotic prescribing practices for hospital inpatients. Cochrane Database Syst Rev. 2017;2:CD003543. 28178770 10.1002/14651858.CD003543.pub4 PMC6464541

[3] Goodman KE , Pineles L , Magder LS , et al. Electronically available patient claims data improve models for comparing antibiotic use across hospitals: results from 576 U.S. facilities. Clin Infect Dis 2020;5:e4484–e4492. 10.1093/cid/ciaa1127 PMC8662758 32756970

[4] Linder J , Meeker D , Fox C , et al. Durability of Benefits of Behavioral Interventions on Inappropriate Antibiotic Prescribing in Primary Care: follow-up from a cluster randomized clinical trial. Open Forum Infect Dis 2016;3(suppl_1):S1.

[5] Allen JM , Dunn R , Bush J. Effect of prescriber peer comparison reports on fluoroquinolone use across a 16-facility community hospital system. J Am Coll Clin Pharm 2019;2:502–508.

[6] Burstin H , Leatherman S , Goldmann D. The evolution of healthcare quality measurement in the United States.J Intern Med 2016;279:154–159. 26785953 10.1111/joim.12471

[7] Meddings JA , Reichert H , Hofer T , McMahon LF. Hospital report cards for hospital-acquired pressure ulcers: how good are the grades? Ann Intern Med 2013;159:505–513. 24126644 10.7326/0003-4819-159-8-201310150-00003 PMC3832180

[8] Ranganathan M , Hibbard J , Rodday AMC , et al. Motivating public use of physician-level performance data: an experiment on the effects of message and mode.Med Care Res Rev 2009;66:68–81. 18923193 10.1177/1077558708324301

[9] Scholle SH , Roski J , Adams JL , et al. Benchmarking physician performance: reliability of individual and composite measures.Am J Manag Care. 2008;14:833–838. 19067500 PMC2667340

[10] Onwubiko UN , Mehta C , Wiley Z . Derivation of a risk-adjusted model to predict antibiotic prescribing among hospitalists in an academic healthcare network. Antimicrob Steward Healthc Epidemiol 2024;4:e163. 39411663 10.1017/ash.2024.422 PMC11474874

